# *Lactobacillus reuteri* strains protect epithelial barrier integrity of IPEC‐J2 monolayers from the detrimental effect of enterotoxigenic *Escherichia coli*

**DOI:** 10.14814/phy2.13514

**Published:** 2018-01-25

**Authors:** Shokoufeh Karimi, Hans Jonsson, Torbjörn Lundh, Stefan Roos

**Affiliations:** ^1^ Department of Molecular Sciences Uppsala BioCenter Swedish University of Agricultural Sciences Uppsala Sweden; ^2^ Department of Animal Nutrition and Management Swedish University of Agricultural Sciences Uppsala Sweden

**Keywords:** Enterotoxigenic *Escherichia coli* (ETEC), IPEC‐J2, *Lactobacillus reuteri*, mucosal integrity

## Abstract

*Lactobacillus reuteri* is an inhabitant of the gastrointestinal (GI) tract of mammals and birds and several strains of this species are known to be effective probiotics. The mechanisms by which *L. reuteri* confers its health‐promoting effects are far from being fully understood, but protection of the mucosal barrier is thought to be important. Leaky gut is a state of abnormal intestinal permeability with implications for the pathophysiology of various gastrointestinal disorders. Enterotoxigenic *Escherichia coli* (ETEC) can invade the intestinal mucosa and induce changes in barrier function by producing enterotoxin or by direct invasion of the intestinal epithelium. Our hypothesis was that *L. reuteri* can protect the mucosal barrier, and the goal of the study was to challenge this hypothesis by monitoring the protective effect of *L. reuteri* strains on epithelial dysfunction caused by ETEC. Using an infection model based on the porcine intestinal cell line IPEC‐J2, it was demonstrated that pretreatment of the cells with human‐derived *L. reuteri* strains (ATCC PTA 6475, DSM 17938 and 1563F) and a rat strain (R2LC) reduced the detrimental effect of ETEC in a dose‐dependent manner, as monitored by permeability of FITC‐dextran and transepithelial electrical resistance (TEER). Moreover, the results revealed that ETEC upregulated proinflammatory cytokines IL‐6 and TNF *α* and decreased expression of the shorter isoform of ZO‐1 (187 kDa) and E‐cadherin. In contrast, pretreatment with *L. reuteri* DSM 17938 and 1563F downregulated expression of IL‐6 and TNF *α*, and led to an increase in production of the longer isoform of ZO‐1 (195 kDa) and maintained E‐cadherin expression. Interestingly, expression of ZO‐1 (187 kDa) was preserved only when the infected cells were pretreated with strain 1563F. These findings demonstrate that *L. reuter*i strains exert a protective effect against ETEC‐induced mucosal integrity disruption.

## Introduction

Leaky gut takes place when the intestinal barrier is damaged and enteritis occurs. This condition has been implicated in the pathophysiology of a variety of gastrointestinal disorders, diseases, and autoimmunity (Fasano 2012). The intestinal epithelium provides a highly regulated barrier against luminal antigenic contents, microbes, and free diffusion of solutes (Ulluwishewa et al. 2011). The mucosa is a multilayer barrier, including physical and immunological defenses (Scaldaferri et al. 2012), where the physical barrier is formed by mucus and a single epithelial cell layer. The intracellular space between the cells is sealed by junctional complexes, including tight junctions (TJ) and adherens junctions (AJ), which are important for cell‐cell contact (Hartsock and Nelson 2008). E‐cadherin and ZO‐1 are two important members of junctional complexes and are both contributing to mucosal integrity. E‐cadherin is the major adherens junction protein that indirectly regulates the organization of the actin cytoskeleton and ZO‐1 is a cytoskeleton connector (Hartsock and Nelson 2008) and scaffolding protein between cytoplasmic and transmembrane proteins (Utepbergenov et al. 2006). ZO proteins closely link with tight junctions (TJ) as part of the TJ complex. (The junctional complexes are involved in the para‐cellular pathway and in facilitating influx of ions and solutes between cells.) (Assimakopoulos et al. 2011). There has been growing interest in studying ZO‐1 and E‐cadherin proteins due to their critical role in the structural and functional organization of the junctional complexes and subsequent mucosal barrier establishment and stability (Assimakopoulos et al. 2011; Gehren et al. 2015; Bardag‐Gorce et al. 2016; Costanzo et al. 2016; Lee et al. 2016).

The intestinal mucosal immune system and its mediators act as an immunological barrier and enhance mucosal barrier function. It is not known whether a leaking mucosal barrier causes dysregulation of the mucosal immune system, including upregulation of proinflammatory cytokines (Fasano and Shea‐Donohue 2005; Lee 2015), or whether cytokines produced by different cell types modulate intestinal permeability and leakage (Clayburgh et al. 2004; Al‐Sadi et al. 2009). However, there is a strong link between upregulation of the proinflammatory cytokines and disruption of the intestinal barrier.

Among many pro‐ and anti‐inflammatory cytokines that orchestrate the immune response, tumor necrosis factor alpha (TNF*α*) and interleukin‐6 (IL‐6) are key players in barrier function. In this study, these two cytokines were chosen to investigate mucosal barrier function because they are predominant cytokines and are elevated in most inflammatory conditions (Scheller et al. 2011) and because they act on the intestinal epithelium and modulate epithelial TJ permeability (Al‐Sadi et al. 2009). Despite their recognized importance, their mechanisms of action on intestinal barrier are not well known and, in particular, the effect of IL‐6 on the mucosal barrier is unclear. IL‐6 is a pluripotent cytokine, exhibiting both pro‐ and anti‐inflammatory effects (Scheller et al. 2011) and has been shown to have both protective and destructive effects on the mucosal barrier (Al‐Sadi et al. 2009).

Host‐microbial interactions may result in dysregulation of the cell junction and the mucosal immune system. Enteric pathogens such as enterotoxigenic *Escherichia coli* (ETEC) can cause severe infections in humans and animals and are capable of producing enterotoxins (Handl et al. 1988) that can abolish intestinal mucosal barrier functions. They adhere to epithelial cells, colonize the small intestine and cause destructive changes in physiological functions of the intestinal epithelium (Croxen and Finlay 2010). They alter the organization and expression of the junctional proteins and cause activation of inflammatory cascades. These changes lead to a severe increase in mucosal permeability and stimulation of electrolyte and fluid secretion (Berkes et al. 2003). The consequences of these alterations are manifested in symptoms such as diarrhea and dehydration, which is the second leading cause of death in pediatric patients in developing counties (Cheng et al. 2005; Qadri et al. 2005). In addition, ETEC infection is a major cause of traveler's diarrhea and is also considered as a serious threat to farm animal welfare and to the farming industry (Dupont 1995).

Expression of junctional proteins such as ZO‐1 and E‐cadherin has been shown to be regulated by probiotics. Probiotics can affect the mucosal barrier by different mechanisms and may arm the host against the effect of pathogenic bacteria on mucosal integrity (Liu et al. 2015; Walsham et al. 2016; Wu et al. 2016). However, the mechanisms by which probiotics exert their beneficial effects on the host are not well known. Enhancement of mucosal integrity via alteration of TJ and AJ is one of several proposed mechanisms by which certain probiotics may confer a beneficial effect (Zyrek et al. 2007; Anderson et al. 2010; Karczewski et al. 2010).

*Lactobacillus reuteri* is a natural inhabitant of the gastrointestinal tract of mammals and birds (Walter et al. 2011). Strains of *L. reuteri* produce biologically active compounds that modulate host mucosal immunity, such as the molecule histamine (Thomas et al. 2012), antimicrobial compounds such as lactic acid and hydrogen peroxide (Martinez et al. 2009) and reuterin (Chung et al. 1989) that can modulate the host microenvironment and subsequently confer health benefits to the host (Walter et al. 2011).

The interaction between ETEC and *L. reuteri* has been studied previously and a protective effect of a porcine‐derived *L. reuteri* on mucosal permeability using the IPEC‐J2 cell line has been reported (Liu et al. 2015). In one study, downregulation of IL‐8 KC/GRO and IFN‐*γ* was observed in newborn rat pups fed a formula containing LPS and any of the *L. reuteri* strains ATCC PTA 4659, ATCC PTA 5289, and ATCC PTA 6475 (Liu et al. 2010). In addition, enhancement of E‐cadherin, occludin, and ZO‐1 expression and downregulation of proinflammatory cytokine expression of TNF*α* and IL‐6 in newborn piglets treated with *L. reuteri* I5007 has been reported recently (Yang et al., 2015).

The aim of this study was to challenge the hypothesis that *L. reuteri* can protect epithelial barrier integrity of polarized IPEC‐J2 monolayers from the detrimental effect of ETEC. The goal was also to reveal any possible protective mechanism by following the expression of tight junction and adherens junction proteins and proinflammatory cytokines.

## Material and Methods

### Bacterial strains and growth conditions

The strains used in this study are listed in Table 1. *Lactobacillus reuteri* strains were cultured overnight at 37°C in de Man Rogosa Sharpe (MRS) broth (Oxoid, Basingstoke, Hampshire, UK). ETEC strain 853/67 was cultivated in Luria‐Bertani (LB) broth (Sigma Aldrich, Saint Louis, MI, USA) overnight at 37°C with vigorous shaking at 150 rpm. ETEC strain 853/67 is a porcine clinical isolate capable of producing at least three different types of enterotoxins: LT, STI, and STII (Handl et al. 1988). The bacterial cultures were harvested by centrifugation for 5 min at 2800 g and washed once with phosphate‐buffered saline (PBS, pH 7.4). After washing, the bacterial pellets were resuspended in antibiotic‐free cell culture medium to the desired concentration (OD_600_ = 0.1) prior to treatment of the IPEC‐J2 cells. The bacteria were counted using a Helber bacteria counting chamber under a light microscope. The IPEC‐J2 cells were fed with antibiotic‐free medium and 1% FBS at 16 h prior to experiments. The IPEC‐J2 cells were either infected directly with ETEC with the dose 5x10^6^ bacteria per cell culture insert (1.12 cm^2^), corresponding to a multiplicity of infection (MOI) of 10 per cell, or first pretreated with *L. reuteri* strains with doses of 5 × 10^7^ and 5 × 10^8^ bacteria per cell culture insert, corresponding to a multiplicity of bacteria (MOB) of 100 and 1000 bacteria per seeded cell, respectively. In the pretreatment, the cells were pretreated with *L. reuteri* strains DSM 17938, ATCC PTA 6475, 1563F or R2LC (Table 1) for 6 h and then washed twice with PBS and infected with ETEC for 4 or 6 h. All bacteria were added to the apical pole of the cell culture.

**Table 1:** Bacterial strains used in this study

Strains	Description	Reference, source
*Lactobacillus reuteri* ATCC PTA 6475	ATCC PTA 6475 (previously designated MM4‐1A), isolated from human breast milk.	A kind gift from BioGaia AB, Stockholm, Sweden
*Lactobacillus reuteri* R2LC	R2LC, isolated from rat intestine.	A kind gift from Siv Ahrné, Lund University, Sweden Ahrné et al. (1992)
*Lactobacillus reuteri* DSM 17938	DSM 17938 is the daughter strain of *L. reuteri* ATCC 55730, isolated from breast milk.	Rosander et al. (2008). A kind gift from BioGaia AB, Stockholm, Sweden
*Lactobacillus reuteri* 1563F	1563F, **i**solated from faeces of a healthy woman. Also named DSM 27131.	A kind gift from BioGaia AB, Stockholm, Sweden. First described in this study.
Enterotoxigenic *Escherichia coli* (ETEC); strain 853/67	Porcine clinical isolate which exhibits the LT, STI, STII, and K88 + phenotype.	Handl et al. (1988).

For measurements of TJ protein expression and inflammatory cytokine, IPEC‐J2 cells in each well were pretreated with 100 MOB of *L. reuteri* strains for 6 h, then washed twice with antibiotic‐free medium and infected with 10 MOI of ETEC for 4 h.

### Epithelial cell culture (IPEC‐J2 cell line)

The porcine jejunal epithelial IPEC‐J2 cell line (kindly provided by Nguyen Lien Thi Minh and Kerstin Skovgaard, Technical University of Denmark, National Veterinary Institute, Denmark) was used for the experiments. IPEC‐J2 is a jejunal epithelial cell line derived from cells isolated from a neonatal piglet less than 12 h old. The cell line was originally derived from a single animal and maintained as a culture (Berschneider 1989) and characterized by several methods as an in vitro infection model (Schieracket al. 2006).

IPEC‐J2 cells were grown and maintained in complete Dulbecco's modified Eagle's medium (DMEM)/F‐12 Ham containing 0.12% sodium bicarbonate, 15 mmol/L HEPES, pyridoxine and L‐glutamine (Sigma Aldrich), supplemented with Pen/Strep (penicillin, 100 U/mL and streptomycin, 100 *μ*g/mL), 5% heat‐inactivated fetal bovine serum (FBS; Sigma Aldrich) and 0.5 mmol/L sodium pyruvate, and incubated at 37°C in an atmosphere of 5% CO_2_. The medium was changed every other day. The cell cultures were tested by polymerase chain reaction (PCR) and determined to be free of mycoplasma contamination.

### Trans‐epithelial electrical resistance (TEER) measurement

To determine the effect of *L. reuteri* and ETEC on the integrity of the IPEC‐J2 cell monolayers, 5 × 10^5^ IPEC‐J2 cells were seeded on transwell filters with area 1.12 cm^2^ and 0.4 *μ*m pore size and cultured in medium containing 5% FBS for 10–12 days to allow polarization. It has earlier been shown that IPEC‐J2 cells are differentiated into tight monolayers after 10 days of culturing (Geens and Niewold 2011). A high seeding density was used in order to saturate the available area for attachment and avoid cell proliferation (Cereijido et al. 1978). Trans‐epithelial electrical resistance (TEER) was measured by a Millicell Electrical resistance System (Millipore). Prior to the experiments, TEER values were obtained every day and during the experiments with *L. reuteri*/ETEC, TEER was measured after 0, 2, 4, and 6 h. Measurements were corrected for the resistance of blank filters and for the membrane area.

We achieved a stable TEER within 10–12 days post seeding. Based on a prestudy, cell monolayers with TEER values between ~ 4000 and ~10,000 Ω cm^2^ were used. Monolayers with TEER values below ~ 4000 were discarded due to a high permeability of FITC‐dextran. Cells within passage 5–10 were used in all experiments.

### Mucosal permeability measurement using FITC‐dextran

In order to quantify the paracellular permeability of monolayers, 1 mg/mL of 4 kDa fluorescein isothiocyanate‐dextran (FITC‐dextran; Sigma) was added to the apical side of the inserts. The basolateral medium aliquots were measured after 6 h of incubation. The diffused fluorescent tracer was then measured by fluorometry (excitation, 492 nm; emission, 520 nm) using a FLUOstar Omega Microplate Reader (BMG Labtech, Ortenberg, Germany).

### Extraction of protein and immunoblotting

For assays concerning TJ protein and inflammatory cytokine expression, IPEC‐J2 cells were cultured in six‐well plates for complete confluence, at the equivalent of approximately 1.5 × 10^6^ cells per well. Total protein was extracted from the cells using lysis buffer from the mirVana PARIS Kit (Ambion, Thermo Fisher Scientific, Austin, TX, USA). Adhered cells in each well were scraped from the culture dish (using a rubber scraper) into 300 *μ*L of ice‐cold lysis buffer containing protease inhibitor and then incubated on ice for 10 min according to the manufacturer's instructions. The lysed cells were centrifuged at 10,000*g* for 5 min at 4°C and the supernatant was collected. The total protein concentrations were measured using a Qubit 3.0 Fluorometer with a protein assay kit (Invitrogen, Thermo Fisher Scientific, Waltham, MA, USA). Equal amounts of protein (40 *μ*g) and protein standard marker were loaded on 4–15% precast SDS‐polyacrylamide gels (PAGE) using a mini‐gel apparatus (Bio‐Rad Laboratories, Hercules, CA, USA), in order to separate the proteins by electrophoresis. The proteins were then transferred to Immune‐Blot polyvinylidene difluoride (PVDF) membranes (Bio‐Rad) using a wet electrotransfer method.

The membrane was blocked in 5% skim milk (Merck, Darmstadt, Germany) in PBS containing 0.05% Tween 20 (PBS‐T) at room temperature (RT) for 1 h and incubated with agitation overnight at 4°C with the following primary antibodies: rabbit polyclonal anti‐cadherin‐1 (antibodies‐online, Atlanta, GA, USA), 1/2000; anti‐ZO‐1 (Abcam, Cambridge, UK), 1/100; and anti‐*β*‐actin (LSBio, Seattle, WA, USA), 1/1000. The reference protein *β*‐actin was used as loading control. The membranes were then washed three times for 10 min and incubated for 1 h at RT with HRP‐conjugated goat anti‐rabbit sera (Thermo Fisher), 1/20,000. The immunoblots were washed four times with PBS‐T for 10 min at RT. The protein bands were developed using Amersham ECL Prime Western Blotting Detection Reagent (GE Healthcare, Little Chalfont, Buckinghamshire, UK) according to the manufacturer's recommendation and visualized by chemiluminescence. Band densities were quantified using Image Lab 5.2.1 software for total protein normalization.

### RNA isolation and expression analysis by RT qPCR

IPEC‐J2 cells (1.5 × 10^6^) were seeded in six‐well plates 24 h prior to the experiment. Cells were pretreated with 100 MOB of *L. reuteri* strains ATCC PTA 6475, DSM 17938 and 1563F for 6 h prior to infection with 10 MOI of ETEC for 4 h. The total RNA of each sample was isolated from the IPEC‐J2 cells using a NucleoSpin RNA II Kit (Macherey‐Nagel, Düren, Germany) according to the manufacturer's instructions. RNA integrity was determined by gel electrophoresis and by OD_260_/OD_280_ nm absorption ratio >1.8 using a Qubit 3.0 Fluorometer (Invitrogen) and a Qubit RNA BR kit (Molecular probes, Life Technologies, USA). Approximately 1 *μ*g RNA was reverse‐transcribed to cDNA using SuperScript IV First‐Strand Synthesis System (Invitrogen, Thermo Fisher Scientific) according to the manufacturer's instructions. For qPCR reaction, a master mix was prepared as follows: 1 *μ*L of forward and reverse primer (0.4 *μ*mol/L) for target/reference genes listed in Table 2, 10 *μ*L of Maxima SYBR Green/ROX qPCR (2x) (Thermo Fisher, USA), 1 *μ*L of cDNA and 7 *μ*L of double‐distilled water in a final volume of 20 *μ*L. The following PCR protocol was used: amplification and quantification program repeated 40 times (94°C for 60 sec, different annealing temperature (53 and 54.5°C) for 60 sec based on gene‐specific primer sequences, 72°C for 60 sec, melting curve program (60–95°C with a heating degree of 0.1°C/sec and a continuous fluorescence measurement). The qPCR was performed using a Thermal Cycler real‐time PCR system, CFX‐96 (Bio‐Rad). The relative gene expression was quantified in triplicate for each sample. No primer‐dimers or nonspecific product were generated during the 40 amplification cycles applied. The relative expression ratio of IL‐6 and TNF*α* was calculated based on the real‐time PCR efficiencies and the Ct deviation of treated samples compared with the control (untreated) and normalized against the expression level of the *β*‐actin. The calculations were performed using Pfaffl method.

**Table 2:** Primers used in this study

Gene	Primer	Sequence (5′‐3′)	Product size (bp)	TM
TNF*α*	Forward	ATTCAGGGATGTGTGGCCT	120	58
	Reverse	CCAGATGTCCCAGGTTGC		
IL‐6	Forward	TGGATAAGCTGCAGTCACAG	109	54
	Reverse	ATTATCCGAATGGCCCTCAG		
*β*‐actin	Forwards	TGCGGGACATCAAGGAGAAG	216	60
	Reverse	AGTTGAAGGTGGTCTCGTGG		

### Statistical analyses

All experiments were performed in triplicate or quadruplicate independent seedings, with three technical replicates in each. Statistical differences between all groups of treatments for TEER and FITC‐dextran measurements were assessed using one‐way ANOVA, followed by a Student's multiple *t*‐test. Statistical analyses for real‐time PCR and immunoblotting experiments were conducted with a one‐way ANOVA analysis, followed by a Tukey's multiple comparisons post‐test and a Student's multiple *t*‐test, respectively. Differences were considered statistically significant at *P *≤* *0.05 and presented as mean ± SEM. All statistical analyses were performed with the JMP Pro 11 statistical software.

## Results

### Protective effect of *L. reuteri* on the epithelial barrier

**Figure 1:**
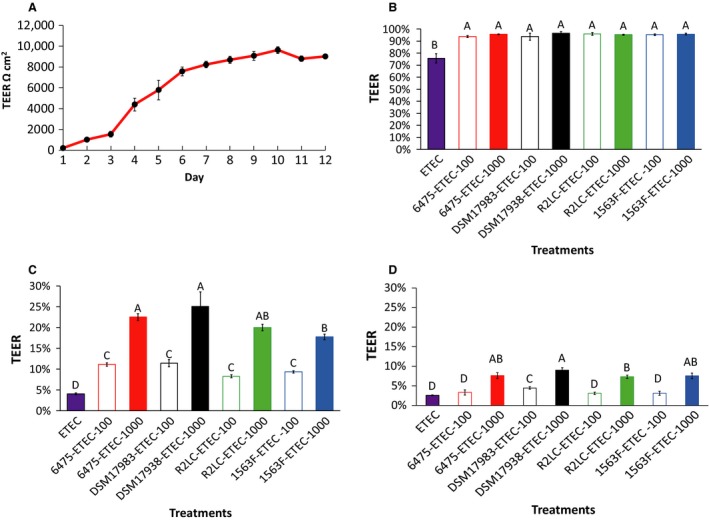
Effect of *Lactobacillus reuteri* strains on the deleterious effect of ETEC on transepithelial electrical resistance (TEER). The IPEC‐J2 cells were grown on transwell filters for 12 days and TEER was measured every day. A representative experiment of progression of TEER values (A). Polarized monolayers were pretreated or not pretreated with *L. reuteri* strains ATCC PTA 6475, DSM 17938, R2LC and 1563F at multiplicity of bacteria (MOB) of 100:1 and 1000:1 for 6 h. After 6 h exposure, *L. reuteri* was washed off and the monolayer was infected with ETEC at multiplicity of infection (MOI) 10 for 6 h. TEER measurement, 2 h postinfection (B). TEER measurement, 4 h postinfection (C). TEER measurement, 6 h postinfection (D). TEER was measured in ohms and corrected for the resistance of blank filters and for the membrane area and expressed as percentage of the starting value. Data are given as means (±SEM) three‐four independent seedings. Columns with different letters are significantly different (*P* ≤ 0.05).

Polarized monolayers of IPEC‐J2 cells were established with stable TEER after 10–12 days of cultivation on transwell filters (a representative experiment is shown in Fig. 1A). Mono‐incubation of the monolayers with high (1000:1 MOB) and low (100:1 MOB) doses of the *L. reuteri* strains gave no significant alterations of TEER after 2, 4 and 6 h incubation compared with the control (Fig. S1). Likewise, flow through of the tracer FITC‐dextran after 6 h of treatment with *L. reuteri* did not differ from the control.

The challenge with ETEC induced a reduction in TEER in a time‐dependent manner (Fig. 1 B–D), and all *L. reuteri* strains were partly able to protect the epithelial monolayer from this challenge. Two hours post ETEC challenge, TEER declined by 5% for the *L. reuteri*‐ETEC groups and by 24% for the group pretreated without lactobacilli. Four and 6 h postinfection, the decline for the ETEC group reached 96% and 97%, respectively, and pretreatment with the *L. reuteri* strains continued to delay of the ETEC‐induced damage to the IPEC‐J2 monolayers. A dramatic drop in resistance from 2 to 4 h post‐infection was observed, though still the monolayer treated with both low and high doses of *L. reuteri* showed significantly higher TEER compared to the ETEC treated group (*P *<* *0.05). The TEER values were significantly higher (by two‐fold) for the higher dose compared with the low‐dose treatment. In addition, only marginal differences between the four *L. reuteri* strains were observed (Fig. 1 B–D).

**Figure 2:**
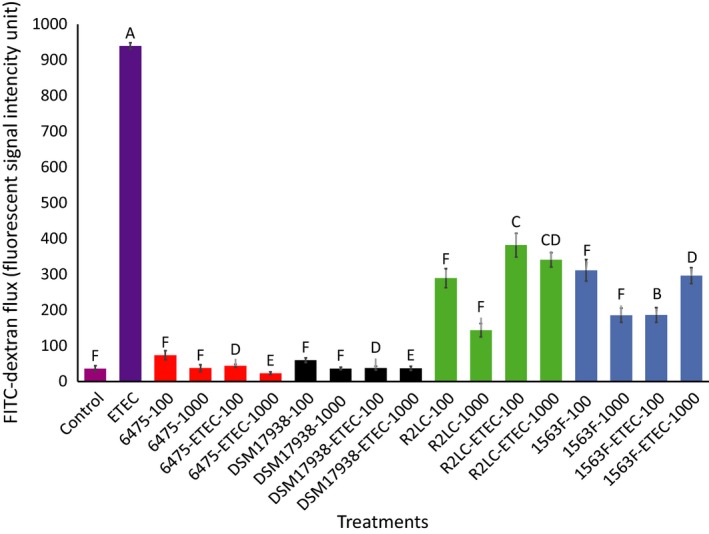
Effect of *Lactobacillus reuteri* strains on FITC‐dextran permeability enhanced by ETEC. Monolayers were pretreated with four strains of *L. reuteri* at MOB 100 and 1000 for 6 h, half of them were challenged with ETEC for 6 h, and finally 1 mg/mL of FITC‐dextran (4 kDa) was added to the apical side of the inserts. The samples were taken from the basolateral pole 6 h post‐treatment and analysed for fluorescence intensity (excitation, 492 nm; emission, 520 nm). Data given are means (±SEM) of three independent seedings. Values with different letters are significantly different at *P *≤* *0.05.

The protective effect of *L. reuteri* against ETEC damage to the monolayer was also apparent in the FITC‐dextran flux experiments (Fig. 2). Pre‐treatment of the monolayers with high and low doses of the *L. reuteri* strains decreased the leakage of FITC‐dextran by 60–85% and 50–70%, respectively, compared with the ETEC group (Fig. 2). Pretreatment with ATCC PTA 6475 and DSM 17938 was more efficient than pre‐treatment with 1563F and R2LC. Lactobacilli alone did not affect the FITC permeability compare to the control.

### *Lactobacillus reuteri* reverses the deleterious effect of ETEC on expression of adherens and tight junctions

Polarized monolayers of IPEC‐J2 were pretreated with *L. reuteri* strains ATCC PTA 6475, DSM 17938 and 1563F for 6 h followed by infection with ETEC for 4 h. To analyze the protective effect of *L. reuteri* on adherens and tight junction proteins, the expression of E‐cadherin and ZO‐1 was assessed by immunoblotting experiments.

**Figure 3:**
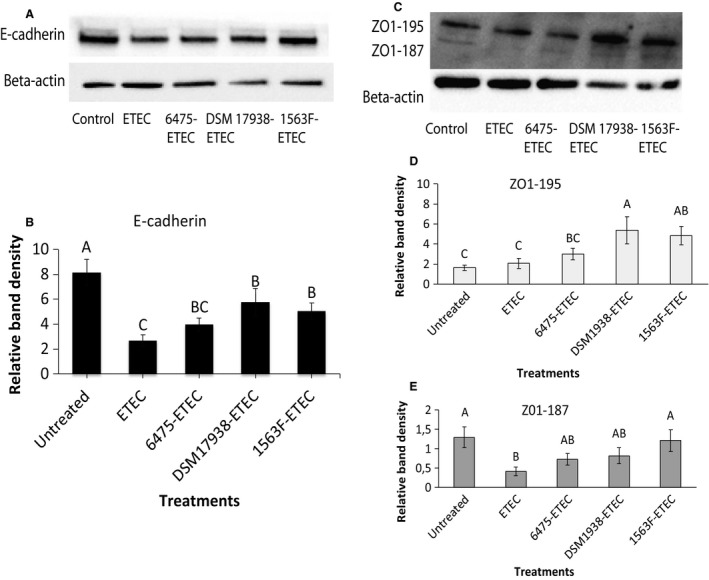
Effect of *Lactobacillus reuteri* strains and ETEC on tight junction protein expression in IPEC‐J2 cells. Cells were grown in six‐well culture plates to a density of 1.5 × 10^6^ cells/well. Polarised monolayers were pretreated with *L. reuteri* strains ATCC PTA 6475, DSM 17938 and 1563F (MOB 100:1) for 6 h and then the monolayers were infected with ETEC (MOI 10:1) for 4 h. The immunoblotting experiments were performed using 40 *μ*g of IPEC‐J2 protein per well. A band of 135 kDa corresponding to E‐cadherin (A). Two bands of 195 kDa and 187 kDa, respectively, corresponding to two different isoforms of ZO‐1, appeared (C). Relative protein levels were quantified using densitometry and expressed as optical density ratio: Relative band densities are shown for E‐cadherin (B), ZO‐1 (195 kDa) (D) and ZO‐1 (187 kDa) (E). The immunoblotting experiments were performed on four independent seedings and the densitometric values of ZO‐1 and E‐cadherin normalized to reference protein, with *β*‐actin as internal standard. Values are mean ± SEM. Columns with different letters are significantly different (*P* ≤ 0.05) as determined by ANOVA.

The expression of E‐cadherin was reduced by more than 60% following ETEC infection of the epithelial cells. Compared with the ETEC treated cells, the expression was approximately 1.5‐fold higher for cells pretreated with 6475 (not significant) and ~2‐fold higher for DSM 17938‐ and 1563F‐treated cells (*P *<* *0.05) (Fig. 3A and B).

The ZO‐1 immunoblots revealed two bands of 195 and 187 kDa, corresponding to two different isoforms of the protein (Fig. 3C). Cells pretreated with *L. reuteri* followed by treatment with ETEC showed higher expression of the 195 kDa isoform compared to untreated cells and cells only treated with ETEC. The amount of ZO1‐195 kDa was increased 3‐ to 5‐fold for DSM 17938‐ETEC and 1563F‐ETEC compared with the control and ETEC groups (*P *<* *0.05) (Fig. 3D). In the cells infected with ETEC alone, a threefold decline in expression of the shorter isoform ZO1‐187 kDa was observed compared with the untreated control. Among *L. reuteri*‐ETEC groups, cells pretreated with 1563F had a significantly higher expression of ZO1‐187 kDa compared with the ETEC infected group (*P *<* *0.05) (Fig. 3E).

### *Lactobacillus reuteri* ameliorates inflammation induced by ETEC

The IPEC‐J2 cells were cultivated on plates and pretreated with *L. reuteri* for 6 h followed by infection with ETEC for 4 h. To investigate the protective effect of *L. reuteri* with respect to inflammatory response to ETEC infection, the mRNA expression of the inflammatory cytokines TNF*α* and IL‐6 was assessed.

**Figure 4:**
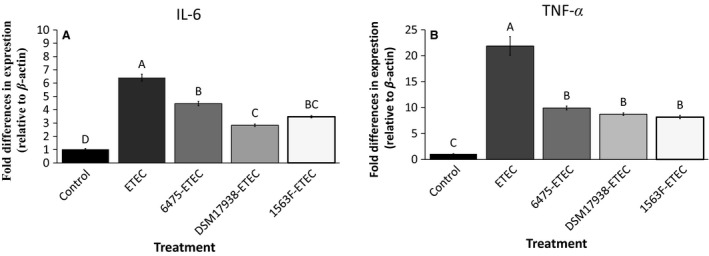
Effect of *Lactobacillus reuteri* strains on ETEC‐induced IL‐6 and TNF *α* expression in IPEC‐J2 cells. IL‐6 expression (A) and TNF *α* expression (B). Cells were grown in six‐well tissue culture plates to a density of 1.5 × 10^6^. Polarized monolayers were pretreated with *L. reuteri* strains ATCC PTA 6475, DSM 17938 and 1563F (MOB 100:1) for 6 h. Thereafter, monolayers were challenged with ETEC (MOI 10:1) for 4 h. The results were considered significant at *P < *0.05, as determined by ANOVA. The data represent results from 3 to 4 independent seedings.

ETEC infection strongly increased the expression of TNF*α* and IL‐6, by 21‐ and ~6‐fold, respectively, compared with the control (*P *<* *0.05; Fig. 4). Pretreatment with all strains of *L. reuteri* significantly inhibited the ETEC induced expression of the cytokines. DSM 17938 showed the best ability to reduce the expression of IL‐6 (*P *<* *0.05) (Fig. 4A), whereas all three strains had the same ability to inhibit TNF*α* expression (Fig. 4B).

## Discussion

Use of probiotics is a promising approach for improving leaky gut and this study examined if *L. reuteri* strains have potential to reduce mucosal leakage and the mechanism behind their beneficial effects. The emphasis was on their ability to protect intestinal enterocytes and mucosal integrity against the enteric pathogen ETEC.

Previous studies have demonstrated that pathogens, including ETEC, can alter the intestinal barrier function through different mechanisms (Philpott et al. 1998; Johnson et al. 2010; Shen et al. 2010). This study confirmed that ETEC disrupts the mucosal barrier and found that it was associated with a strong decrease in expression of E‐cadherin and the shorter isoform of ZO‐1. The decline in TEER and enhanced permeability to FITC‐dextran in response to ETEC has been shown in earlier studies (Liu et al. 2015; Yang et al. 2015). The finding that ETEC downregulates ZO‐1 has also been reported earlier (Zhang et al. 2015; Wu et al. 2016). Two different isoforms of human ZO‐1, with size 195 and 187 kDa, were detected. These are described at UniProt (Entry Q07157, www.uniprot.org/uniprot/Q07157) and the shorter isoform lacks amino acids 922‐1001. To the best of our knowledge no functional differences have been described for the isoforms.

The pretreatment of the intestinal epithelial cells with strains DSM 17938 and 1563F prevented the increase in permeability caused by ETEC, possibly by maintaining E‐cadherin expression and upregulating ZO1‐195. Strain 1563F also attenuated the destructive effect on ZO1‐187 expression and the expression remained as high as for untreated IPEC‐J2 cells. In line with this, upregulation of TJ proteins ZO‐1 (Karczewski et al. 2010) and E‐cadherin in Caco‐2 cells treated with *L. plantarum* has been reported previously (Anderson et al. 2010). It is known that TJ proteins can be used as receptors by enteric pathogens (Berkes et al. 2003). This raises the possibility that *L. reuteri* may prevent epithelial damage by competing with ETEC for binding to TJ proteins.

Several studies have shown that different cytokines can regulate the junctional complex and cytoskeleton structure and function (Nusrat et al., 2000) and TNF*α* and IL‐6 are both linked to increased intestinal permeability (Scheller et al. 2011; Al‐Sadi et al. 2014). The hijacking of cellular molecules and signaling pathways of the host, including upregulation of proinflammatory cytokines, is often part of the pathogenic process (McGuckin et al. 2011). For example, the proinflammatory roles of IL‐6 and TNF*α* have been reported to be linked with ETEC and loss of mucosal permeability (Shimazu et al. 2012; Wang et al. 2016). In agreement with this, our results showed that disruption of the mucosal barrier by ETEC was associated with a strong increase in TNF*α* and IL‐6 expression, which in turn corresponded to a decrease in E‐cadherin and ZO1‐187 expression levels, complete destruction of TEER and enhanced permeability.

IL‐6 has also been described to have another role and protect the mucosal barrier through upregulation of expression of keratin‐8 and keratin‐18 (Wang et al. 2007), however, our results did not support this. We showed that IL‐6 along with TNF‐*α* may contribute to mucosal permeability dysfunction and that the *L. reuteri* strains prevented the increase in expression of both IL‐6 and TNF*α* induced by ETEC. This is in line with results from an earlier study, where *L. reuteri* was shown to downregulate TNF*α* in an in vivo murine colitis model (Peña et al. 2004). An increase in gene expression does not necessarily reflect the release of the corresponding protein. However, it has been shown earlier that upregulation of genes encoding proinflammatory cytokines is associated with release of the corresponding cytokines (Brosnahan and Brown, 2012), which affects the tight junctions and results in mucosal barrier dysfunction (Al‐Sadi et al. 2009).

Our findings suggest that *L. reuteri* prevents an increase in permeability through TJ stabilization and blocking of the proinflammatory response. It is possible that upon downregulation of the elevated expression of the proinflammatory cytokines by *L. reuteri*, the expression of ZO‐1 and E‐cadherin was stabilized, and as a result damage to the intestinal barrier was slowed down, a correlation reported previously in studies conducted in vitro (Tazuke et al. 2003; Ye and Ma 2008; Suzuki et al. 2011) and in vivo (Wang et al. 2001; Yang et al. 2003).

We demonstrated that *L. reuteri* protected the integrity of the enterocyte barrier in a dose‐dependent manner, with a higher dose (1000 MOB) showing better protection of the integrity of epithelial cell junctions. Similarly, the benefit of using a higher dose of a probiotic bacteria in protection of mucosal integrity in an cell culture model has recently been reported (Mokkala et al. 2016). An in vitro study is of course much less complex than a clinical trial, but the results still indicate that the density of the probiotic bacteria in the intestine may influence the clinical outcome. Studies on humans and animals have demonstrated that the beneficial effect of *Lactobacillus* can be dose‐dependent (Fang et al. 2009; Suo et al. 2012; Ouwehand 2017). Using a meta‐analysis approach, an earlier study also demonstrated better efficacy of higher doses of probiotics (greater than 10^11 ^CFU) than lower doses in patients with high blood pressure (Khalesi et al. 2014). In general, the proper dosage of probiotics with regard to safety and effectiveness has not been systematically studied. This is partly due to the high complexity of the gut microbial ecosystem, the heterogeneity of probiotics and the genetic background of the target population. Probiotics can be ineffectual when the optimal dosage for the intended purpose is not applied (Wen et al. 2012).

We have used only one intestinal cell line in our study. However, IPEC‐J2 is to our mind a virtuous choice of model for this type of studies, primarily since it is one of the few cell lines generated from the small intestine. Also, it is nontransformed and nontumorigenic in contrast to cell lines such as Caco‐2 and IEC‐6 (Vergauwen 2015). Differences in expression may occur when cells are grown on plates and transwell filters. However, the results from the expression studies on cells grown on plastic fits well with the changes in permeability of cells grown on filters, implying that the IPEC‐J2 cells likely responds in a similar way during both growth conditions.

In this study we demonstrated the protective effect of the human‐derived *L. reuteri* strain 1563F on mucosal permeability. This is the first investigation of this reuterin‐producing strain, which was originally isolated from a feces sample from an adult.

In conclusion*,* we showed that *L. reuteri* exerts a protective effect on leaky gut induced by ETEC through stabilization of tight junction ZO‐1, adherens junction E‐cadherin and the proinflammatory cytokines TNF*α* and IL‐6. This protective activity might be an important probiotic feature, but needs to be verified in animal models.

## Conflict Of Interest

The authors declare that Stefan Roos is partly employed by BioGaia AB.

## Data Accessibility

## Supporting information




**Figure S1.** Effect of *Lactobacillus reuteri* strains on transepithelial electrical resistance (TEER).
